# The Role of the Wild Boar Spreading African Swine Fever Virus in Asia: Another Underestimated Problem

**DOI:** 10.3389/fvets.2022.844209

**Published:** 2022-04-27

**Authors:** Estefanía Cadenas-Fernández, Satoshi Ito, Cecilia Aguilar-Vega, José Manuel Sánchez-Vizcaíno, Jaime Bosch

**Affiliations:** ^1^VISAVET Health Surveillance Center, Complutense University of Madrid, Madrid, Spain; ^2^Department of Animal Health, Faculty of Veterinary, Complutense University of Madrid, Madrid, Spain

**Keywords:** African swine fever, wild boar, backyard farms, risk areas, Asia

## Abstract

African swine fever (ASF) is a highly lethal infectious disease in naive populations of domestic pigs and wild boar. In Asia, from the first outbreak in August 2018 until the end of November 2021, ASF has been reported in 16 Asian countries. The ASF virus (ASFV) circulation in domestic pigs is considered the main problem in Asia. On the other hand, there are very few reports of ASF in wild boar in this region. However, considering the high wild boar density within the same area of smallholder domestic pig farms in Asia, the occurrence of ASFV infection in wild boar may be underestimated. The role of the wild boar in other ASF epidemiological scenarios, such as Europe, is a key for the maintenance and transmission of the disease. Hence, we performed a preliminary study estimating the extent of ASFV infection in the Asian wild boar population. The potential risk area of ASF-infected wild boar was calculated based on the habitat suitability for wild boar, the kernel density of ASF notification in smallholder farms and wild boar, and the ASFV transmission rate of wild boar. As a result of the analysis, high-, medium-, and low-risk areas were identified throughout Southeast and East Asia. The highest risk area was detected in China, followed by Myanmar, Far East Russia, Thailand, Vietnam, Laos, Cambodia, and the Philippines. Additionally, another risk area was detected from northeastern China to the Korean Peninsula, including Far East Russia. This study shows hot spots where a high risk of infection in wild boar is most likely to occur, helping to control ASF.

## Introduction

African swine fever (ASF) is a highly lethal infectious disease in naive populations of domestic pigs and wild boar (*Sus scrofa*) (1). The control of the disease is hampered by the lack of an effective vaccine and treatment, being limited to strict sanitary measures including the slaughter of affected animals and closure of trade borders, which implies important economic losses (2). Due to the unprecedented spread of the ASF virus (ASFV) in recent years through Europe, Asia, and recently reported in American countries (the Dominican Republic and Haiti; OIE-WAHIS, 2021), it is considered a great challenge currently facing livestock health.

In August 2018, ASFV reached the Asian continent for the first time in history, through China (3). In Asia, the disease has spread faster than in Europe, affecting 13 more countries in the following year, in this order: Far East Russia, Mongolia, Vietnam, Cambodia, Hong Kong, North Korea, Laos, Myanmar, the Philippines, South Korea, Timor-Leste, and Indonesia. Currently, the expansion of ASF continues in Asia and, in the last two years, the disease has been notified in four more countries, namely, India, Malaysia, Bhutan, and Thailand. The disease has even reached Oceania, with cases of ASF reported in Papua New Guinea in 2020. Over this time, more than eight million pigs have been reported slaughtered in Asia because of ASF (4), leading to devastating economic consequences. Furthermore, the fact that the largest producer of pigs in the world (China) is extensively infected (4) puts the other disease-free countries at serious risk. It should be noted that the movement and illegal trade of infected pigs and pork products is traditionally the major risk of transboundary occurrence and transcontinental spread of ASFV (5).

Knowing the situation in Europe, the role of wild boars in the maintenance and transmission of ASFV should not be underestimated. When wild boar populations are extensively infected, the disease control is limited (6), and represents a constant risk for the domestic pig sector infection (7). Furthermore, the low virulence of ASFV isolates recently reported in China has resulted in chronically infected individuals (8). If chronic infection among wild boar becomes frequent, the disease would be more difficult to control. The disease in wild boar is similar to that observed in domestic pigs—the symptoms can be hyperacute, acute, or chronic, depending on the virulence of the isolate and the host immune system (9). In case of highly virulent isolates in naive animals, hyperacute and acute clinical signs are observed, characterized by a generalized hemorrhagic picture, high fever, and a lethality rate close to 100% (10).

In Asia, the disease is widely reported in domestic pigs, with sporadic notifications in wild boar. Although some Asian countries have included ASF surveillance in wild populations, such as Far East Russia and South Korea, for the rest of the affected countries, the notifications in wild boar remain scarce (11). However, it is estimated that wild boar population abundance is higher in many areas of Asia than in Europe (12). Additionally, in many Asian countries, smallholder farms (backyard farms) are a widespread type of production system, which lack high levels of biosecurity. This scenario allows direct or indirect interaction between wild boar and domestic pigs (13), enabling the transmission of the ASFV between both hosts. All this suggests that ASF is being underdetected in wild boar populations in Asia (14).

For all this, the aim of this study is to estimate the extent of ASFV infection in wild boar populations in Asia. To achieve this objective, we used data on the suitability habitat for wild boar, ASF notifications in wild boar, and the transmission rate of ASF in wild boar. Following other studies (15, 16), we assumed that the higher risk of wild boar infection exists in the following situations: (i) with a higher density of ASF notifications, (ii) with a shorter distance between the infected individuals, and (iii) with ASF outbreak in smallholder farms which locate inside the suitable habitat of wild boar.

## Materials and Methods

### Study Area

The study area is defined by all the countries of the Asian continent.

### Suitability Habitat for Wild Boar

The distribution of the suitable habitat for wild boar was estimated based on the Quality of Available Habitat (QAH) map developed by Bosch et al. (17). The QAH map is a cartographic tool previously suggested as a potential tool for managing ASF (18, 19). Briefly, it is a standardized distribution map based on global land cover vegetation (GLOBCOVER) that quantifies QAH for wild boar. The QAH map provides seven levels of QAH, namely, (i) 0, “absent”; (ii) 0.1, “unsuitable”; (iii) 0.5, “worst suitable area”; (iv) 1, “suitable areas for food or shelter”; (v) 1.5, “suitable areas for food and shelter, but used mainly for the shelter”; (vi) 1.75, “suitable areas for food and shelter, but used mainly for food”; and (vii) 2, “suitable areas for both food and shelter”. In the current study, the QAH map was used to delimitate the distribution of the suitable habitat of the wild boar population in Asia, and to locate and delimitate the contact between domestic pig farms and the wild boar population.

### Density of ASF Notifications

Data on ASF notifications were gathered from the World Animal Health Information System (OIE-WAHIS portal) from March 2017 to January 2021. The information we entered into the database from the notifications was the following: geographical coordinates, country, species, number of cases, number of susceptible animals, and date of start of the outbreak.

Since many of the affected countries in Asia do not follow a wildlife surveillance program, not only notifications on wild boar have been used, but also those on domestic pigs that present a high risk of infection to wildlife. To define these latter notifications, two requirements were considered: (i) smallholder domestic pig farms, selecting only those farms with less than 50 animals, and (ii) farms inside or close (<5 km) to the suitable habitat of wild boar based on the QAH map. Overall, these requirements refer to areas with potential direct or indirect contact between wild boar and domestic pigs.

These ASF notifications were transformed into a grid density map (outbreak density map) based on kernel smoothing techniques estimations (ESRI, Redlands, CA, USA) using ArcGIS 10.4, with a range from 0 to 2,069. According to Pfeiffer et al. (20), the transmission of infectious disease is more likely if the at-risk individuals are close to spatiotemporal windows. Therefore, expanding the outbreak density from the origin of the notifications generates an influenced area of potential infection (15, 16).

### Potential ASF Dispersal Area

A buffer area was built around the outbreak density map using ArcGIS 10.4 to delimitate the potential spread distribution of the disease in wild boar. The bandwidth of the buffer was calculated considering the spreading speed of the disease and the time it has been present in Asia.

In concrete terms, it has been assumed the spreading speed of ASF in wild boar in the Russian Federation, since it is the closest country to the study area from which this information has been previously studied, is 350 km per year (21). The annual spreading speed has been multiplied by the 3 years of ASF presence in Asia to finally calculate the buffer area [350 km × 3 years = 1,050 km (radius buffer area 525 km)].

### Predicting the Risk of ASFV Infection

**Figure 1:**
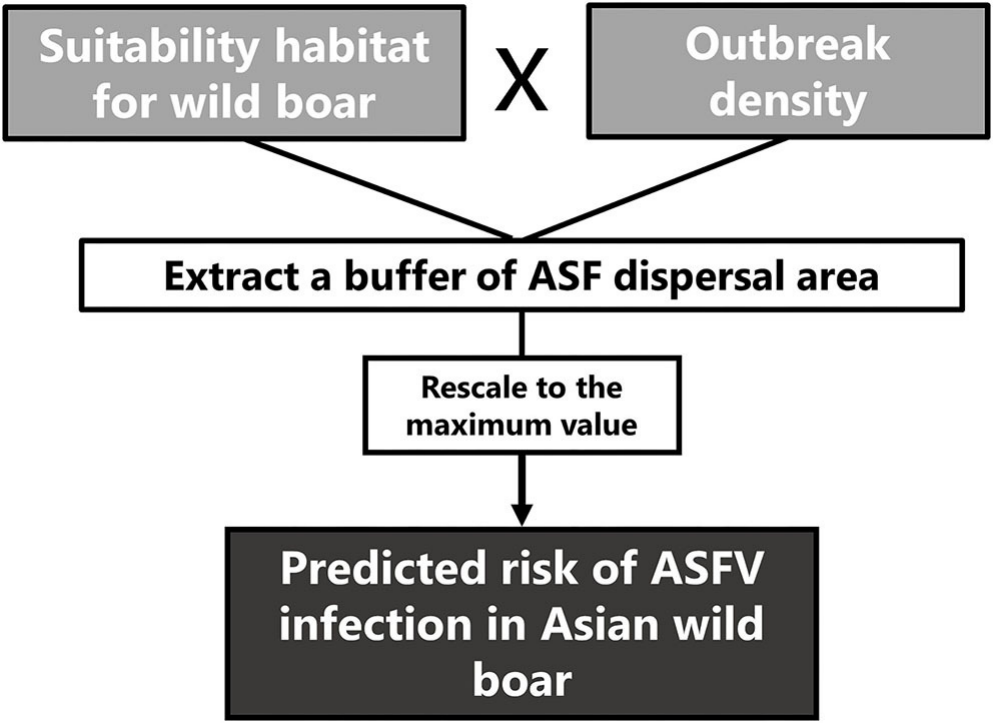
Scheme of the procedure for predicting the risk of African swine fever virus (ASFV) in wild boar in Asia.

To predict the risk of ASFV infection in wild boar across Asia, the values of the outbreak density were multiplied by the habitat suitability for wild boar (QAH categories). The spatial resolution was 1 km^2^. The quantitative risk of the ASFV infection area was extracted using the previously defined buffer to delimitate the ASF dispersal area (Figure 1). Then, the obtained values were rescaled adjusting the risk rank on a scale of 0 to 1. The risk areas were classified into three categories (low, medium, and high risk) based on Jenks' natural break grading method (22). Thus, the sum values of the risk from 0 up to and including 90.2 were considered low risk, the values from 90.3 up to and including 532.6 were considered medium risk, and from 532.7 up to and including 3,049.6 were considered high risk. Finally, for calculating the risk of ASFV infection by country, it was obtained the sum of the risk values of the spatial cells of each country and used the same risk classification.

## Results

**Figure 2:**
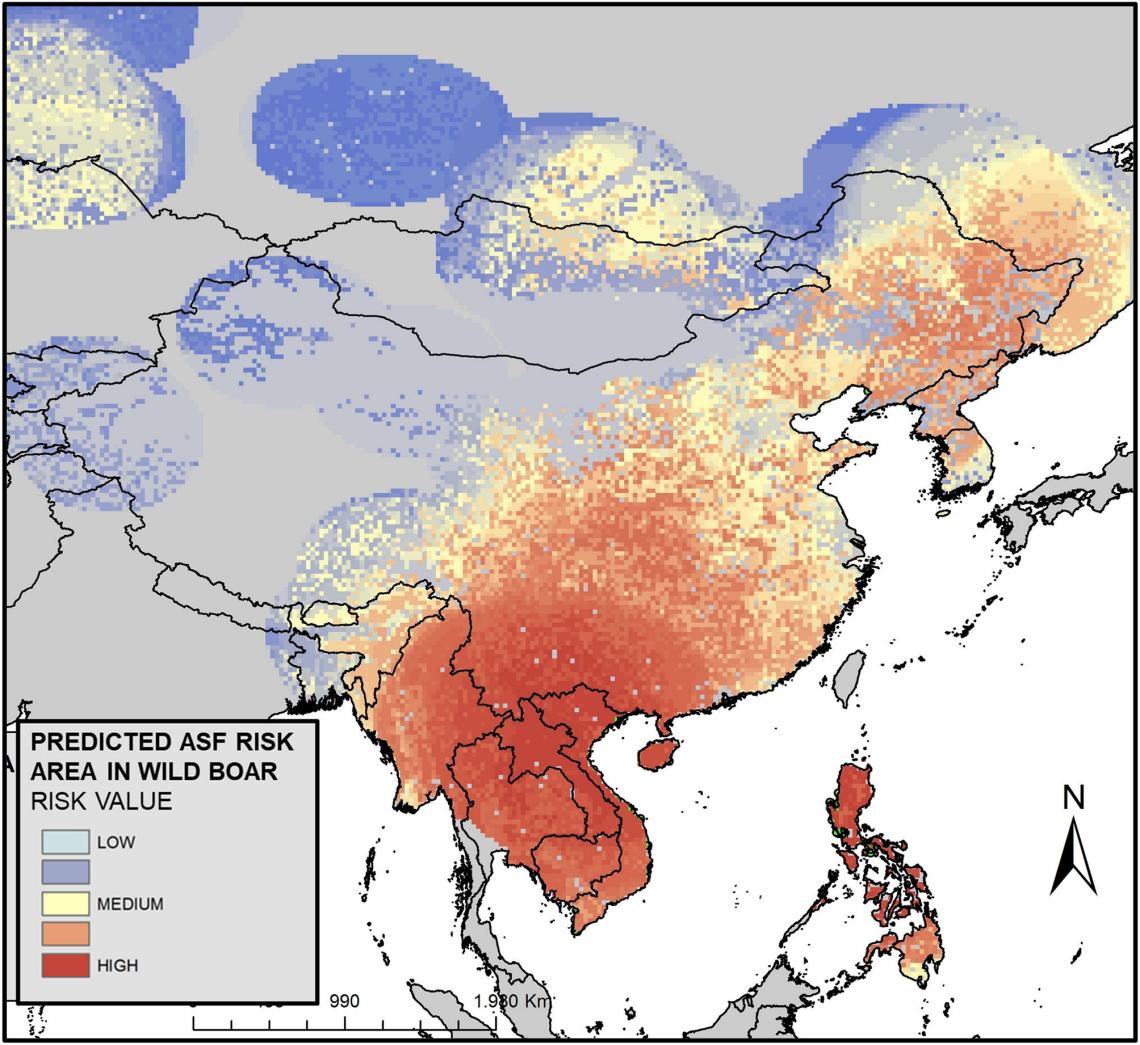
Predicted high-, medium-, and low-risk areas of African swine fever (ASF) in wild boar in Asia (Zoom on the estimated extent of ASF virus infection in wild boar populations in South East and East Asia).

The predicted risk of ASFV infection in wild boar across Asia was spatially quantified and classified into three risk categories: low, medium, and high, as shown in Figure 2 (zoom to South East and East Asia) and in Supplementary Figure S1. The model output shows that southeast Asia concentrates the highest risk areas. However, another major risk area has also been detected from northeastern China to the Korean peninsula, including Far East Russia. Additionally, the model performance is shown in the Korean peninsula and surrounding areas, as an example, in Supplementary Figure S2. The model shows a total of 25 countries at risk from ASF, and 12 more were affected by January 2021, the end of the selected study period. The countries were classified into three risk categories as shown in Table 1. In this way, China resulted in the highest risk. Countries with medium risk were Myanmar, Far East Russia, Thailand, Vietnam, Laos, Cambodia, and the Philippines. The top five countries in low-risk rank were Mongolia, India, North Korea, South Korea, and Kazakhstan. It is important to take into consideration that this rating may be biased due to the size of each country, as it is based on total risk regardless of the size.

**Table 1:** The prediction of the risk countries of African swine fever virus (ASFV) infection in wild boar in Asia, by country until January 2021.

**Country**	**Report of ASFV infection**	**Surface (km^**2**^)**	**Potential infected surface (km^**2**^)**	**Potential infected area (%)**	**Maximum risk value**	**Sum of risk values**	**Risk category**
China	Reported	9,596,961.1	7,520,128.9	78.4	0.99	3,049.6	High
Myanmar	Reported	749,307.04	689,307.0	92.0	0.94	532.6	Medium
Far east Russia	Reported	6,013,593.1	3,013,593.0	50.1	0.61	459.8	Medium
Thailand	Not reported^a^	579,863.5	479,863.5	82.8	1	447.3	Medium
Vietnam	Reported	389,516.9	369,517.0	94.9	1	330.0	Medium
Laos	Reported	267,765.5	267,765.5	100	1	310.5	Medium
Cambodia	Reported	220,334.4	219,334.4	99.5	0.97	170.6	Medium
Philippines	Reported	377,500.1	277,500.0	73.5	1	168.1	Medium
Mongolia	Reported	1,564,110.1	1,233,060.4	78.8	0.43	90.2	Low
India	Reported	3,402,784.2	402,784.1	11.8	0.64	73.9	Low
North Korea	Reported	120,540.1	108,503.5	90.0	0.61	65.2	Low
South Korea	Reported	100,210.2	82,904.4	82.7	0.52	28.1	Low
Kazakhstan	Not reported	2,724,900.1	272,290.8	10.0	0.21	26.2	Low
Bangladesh	Not reported	149,027.2	141,027.1	94.6	0.39	18.2	Low
Kyrgyzstan	Not reported	181,945.9	161,945.9	89.0	0.10	6.7	Low
Bhutan	Not reported^a^	40,150.2	40,150.0	100	0.37	4.8	Low
Nepal	Not reported	147,181.1	21,700.0	14.7	0.22	2.0	Low
Pakistan	Not reported	883,610.1	83,610.0	9.5	0.10	1.8	Low
Indonesia	Reported	3,031,840.2	31,840.0	1.1	0.28	1.0	Low
Tajikistan	Not reported	143,100.2	61,230.0	42.8	0.10	0.7	Low
Uzbekistan	Not reported	447,400.1	10,152.6	2.3	0.10	0.6	Low
Macao	Not reported	40	10.0	25	0.57	0.6	Low
Timor-Leste	Reported	29,800	19,800.0	66.4	0.04	0.3	Low
Afghanistan	Not reported	652,864	10,942.1	1.7	0.05	0.25	Low
Hong Kong	Reported	1,230	1,200.0	97.6	0.23	0.23	Low
Malaysia^b^	Not reported^a^	329,847	-	-	-	-	-

*The risk classification is shown in three categories: high, medium, and low. Asian countries not included in this table have no results and should be considered as “No risk of AFS natural spread due to wild boar to date”.*

a *Countries that reported ASFV infection after the period of study.*

b *Although Malaysia was included in the study area, Malaysia did not show risk in our model. The introduction of ASF in Malaysia by wild boar movements is unlikely based on our available data*.

## Discussion

This is the first study predicting the quantitative risk of ASF infection of wild boar in Asia. Although there are some limitations, these results indicate that the role of wild boar in the spread of ASFV in Asia is underestimated. In addition, the study provides information on high-risk hot spots of infection and ranks countries by risk, pointing out those that could have a greater extent of infected wild boar population in Asia. As it is shown in Supplementary Figure S2, the ASF notifications in wild boar after January 2021 in the Korean peninsula and surrounding areas coincided with the predicted highest risk areas. These results highlight the good adjustment of the model to the new notifications in wild boar.

Once the virus has spread to wild boar, the disease is not easy to control. In case of South Korea, immediately after cases of ASF were reported for wild boar, the government invested great efforts to fence the entire country to try to control the movement of wild boar, but the disease continued to spread (23). In Lithuania, the first case of ASF was notified in 2014, in wild boar. Despite the enormous efforts made to eradicate the disease, it is now considered endemic in wild boar (7), affecting a large part of the country's territory (24). All this proves how difficult and costly in terms of resources and time it is to eradicate it when the wild boar population is widely infected (25). The main economic problem in the circulation of ASFV in Asia is the infection of domestic pigs. However, considering the possibility that the circulation may continue for a long time, knowing the status of ASF cases in wild boar is of great importance for full ASF control.

Establishing a wildlife surveillance system is not an easy task due to its elevated economic and time-related costs. Therefore, the development of epidemiological studies at a large scale is of extreme importance to predict the expansion of ASF in wild boar populations and to measure its impact. Although this study provides a large-scale prediction, further efforts are needed to thoroughly identify the extent of ASFV infection in wild boar in Asia on a local scale. In this way, the bias of the biological limitations over a large geographical area and the different surveillance efforts could be reduced.

As stated above, one limitation of this study is the sampling bias derived from differences among countries in terms of surveillance and disease reporting. As an example, South Korea is one of the Asian countries with the highest ASF notifications in wild boar (11). However, this country does not reflect such a high risk according to our results. This is because South Korea has only a small wild boar-infected area in the north of the country and also because this is one of the few Asian countries that have implemented a wildlife surveillance program. This suggests that there are countries that may have even a larger infected wild boar population than South Korea in the region. Also, this prediction indicates that countries, which have never notified any cases of ASF neither in domestic pigs nor in wild boar during the study period, are at risk, probably for the high density of notifications from neighboring countries. This is the case of Thailand, which has recently notified the disease in domestic pigs (January 2022) and resulted in this study at medium-risk (the fourth most at risk).

A recently published study has found several ASFV-positive wild boar carcasses in Laos and Vietnam (26). These results suggest that these two countries are likely to have a large ASFV-infected wild boar population, which coincides with our results as they are also medium-risk countries (see Table 1).

The results showed the potential natural spread and speed of the disease in wild boar as of January 2021 and under ecological constraints wild boar movement (i.e., spatiotemporally distance to ASF notifications). The posterior notification of cases in Thailand and Bhutan demonstrates that the methodology used here is useful to highlight areas at risk of ASF natural spread. On the other hand, the model output did not include Malaysia, in which ASF cases were reported later. The first reported outbreak was in Borneo Island with no ASF cases reported in the Indonesian or Bruneian parts of the island. Later, the first cases in mainland Malaysia were more than 1,286 km away from the closer ASF notifications in mainland Asia, which also makes introduction by wild boar movements unlikely in the time frame.

It is important to keep in mind certain limitations of this study when evaluating the results and planning future studies. One of them is that the density of domestic pigs has not been taken into account, as well as the distribution of the type of farm. Another aspect to highlight is that the wild boar distribution map is obtained from the prediction of habitat suitability and not from field data. Although this habitat suitability approach has proved to be very effective in predicting the presence of wild boar, it would be advisable for future local studies to have field data that estimate the occurrence of wild boar and their populations.

The information provided in this study is intended to alert about the epidemiological status of ASF in wild boar in Asia and to recognize high-risk hot spots of infection in wildlife. Our results highlight the necessity of considering wild boar in order to properly tackle the prevention, control, and surveillance of ASF. In addition, this map may serve as a guide for implementing risk-based interventions for improving ASF surveillance programs in wildlife.

## Data Availability Statement

The original contributions presented in the study are included in the article/Supplementary Material, further inquiries can be directed to the corresponding author/s.

## Author Contributions

EC-F, JB, CA-V, and JS-V provided substantial contributions to the conception and design of the work. JB and CA-V performed the data analysis. EC-F, JB, and SI drafted and refined the manuscript. CA-V and JS-V performed a critical reading and revision of the manuscript. All authors have read and approved the manuscript.

## Funding

This research was funded by the H2020 VACDIVA 862874 project. EC-F and CA-V are beneficiaries of a Spanish Government-funded PhD fellowship for the Training of Future Scholars (FPU) awarded by the Spanish Ministry of Education, Culture and Sports.

## Conflict of Interest

The authors declare that the research was conducted in the absence of any commercial or financial relationships that could be construed as a potential conflict of interest.

## Publisher's Note

All claims expressed in this article are solely those of the authors and do not necessarily represent those of their affiliated organizations, or those of the publisher, the editors and the reviewers. Any product that may be evaluated in this article, or claim that may be made by its manufacturer, is not guaranteed or endorsed by the publisher.
