# Supplementary material for: The Role of the Wild Boar Spreading African Swine Fever Virus in Asia: Another Underestimated Problem
Source: Front Vet Sci. 2022 Apr 27;9:844209. doi: 10.3389/fvets.2022.844209 (PMC9093143; doi:10.3389/fvets.2022.844209)
Supplement: Supplementary file 1 [file Data_Sheet_1.PDF]

Supplementary Material

Supplementary Figures

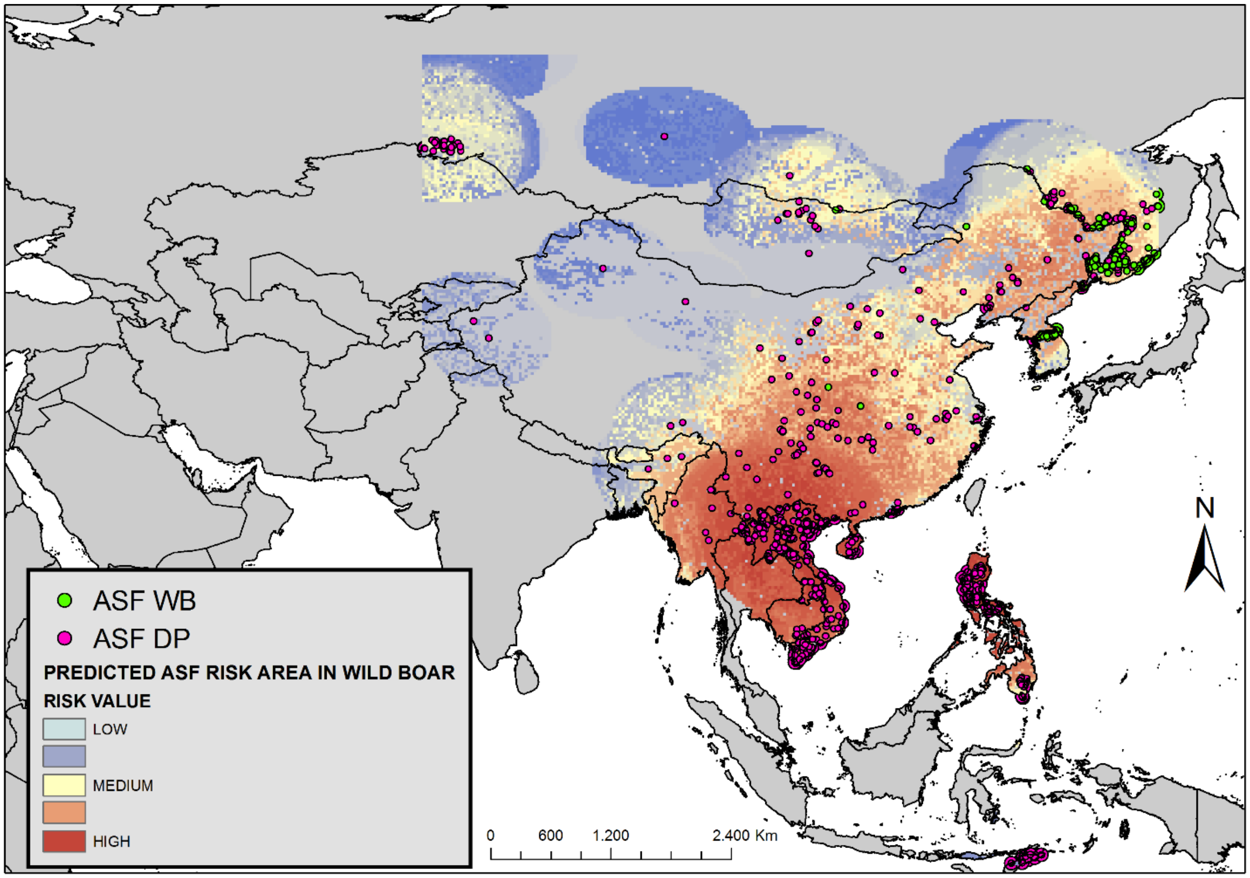

Supplementary Figure 1. Predicted high, medium, and low-risk areas of African swine fever (ASF) in wild boar in Asia. The ASF notifications in wild boar (green dots) and in domestic pigs (pink dot) in Asia during the study period, from March 2017 to January 2021, are shown (OIE-WAHIS portal).

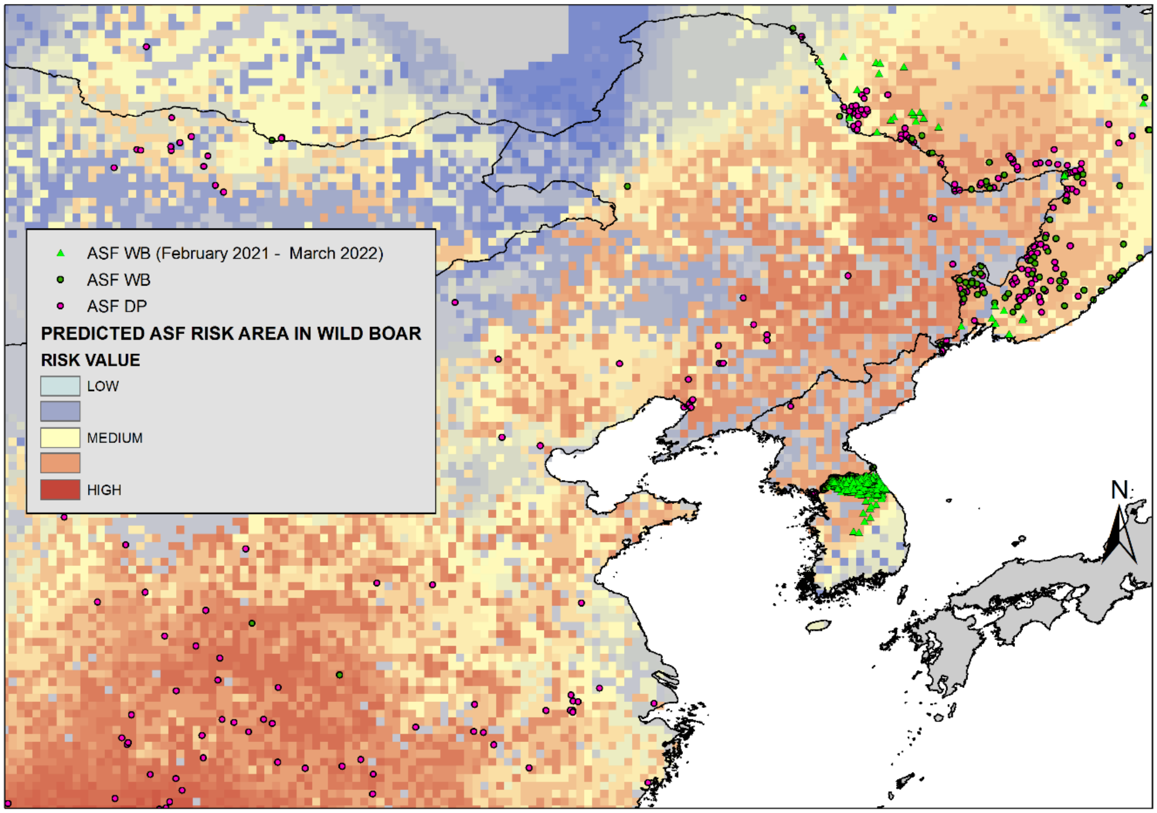

Supplementary Figure 2. Predicted high, medium, and low-risk areas of African swine fever (ASF) in wild boar in the Korean peninsula, far-east Russia and northeastern China. The ASF notifications in wild boar (green dots) and in domestic pigs (pink dots) during the study period, from March 2017 to January 2021, are shown (OIE-WAHIS portal), as well as those notified in wild boar from February 2021 to March 2022 (green triangles; FAO, EMPRES-i).
